# In vitro antimicrobial susceptibility testing methods: agar dilution to 3D tissue-engineered models

**DOI:** 10.1007/s10096-017-3089-2

**Published:** 2017-09-04

**Authors:** A. Schumacher, T. Vranken, A. Malhotra, J. J. C. Arts, P. Habibovic

**Affiliations:** 1 Department of Instructive Biomaterials Engineering, MERLN Institute for Technology-Inspired Regenerative Medicine, Maastricht University, Universiteitssingel 40, Room C3.577, 6229 ER Maastricht, Netherlands; 2 Science and Technology Faculty, University of Twente, Drienerlolaan 5, 7522 NB Enschede, The Netherlands; 3 Department of Orthopaedic Surgery, CAPHRI Care and Public Health Research Institute, Maastricht University Medical Centre, Maastricht, The Netherlands; 4 Orthopaedic Biomechanics Group, Department of Biomedical Engineering, Eindhoven University of Technology (TU/e), Eindhoven, The Netherlands

## Abstract

In the field of orthopaedic surgery, bacterial invasion of implants and the resulting periprosthetic infections are a common and unresolved problem. Antimicrobial susceptibility testing methods help to define the optimal treatment and identify antimicrobial resistance. This review discusses proven gold-standard techniques and recently developed models for antimicrobial susceptibility testing, while also providing a future outlook. Conventional, gold-standard methods, such as broth microdilution, are still widely applied in clinical settings. Although recently developed methods based on microfluidics and microdroplets have shown advantages over conventional methods in terms of testing speed, safety and the potential to provide a deeper insight into resistance mechanisms, extensive validation is required to translate this research to clinical practice. Recent optical and mechanical methods are complex and expensive and, therefore, not immediately clinically applicable. Novel osteoblast infection and tissue models best resemble infections in vivo. However, the integration of biomaterials into these models remains challenging and they require a long tissue culture, making their rapid clinical implementation unlikely. A method applicable for both clinical and research environments is difficult to realise. With a continuous increase in antimicrobial resistance, there is an urgent need for methods that analyse recurrent infections to identify the optimal treatment approaches.

**Graphical abstract:**
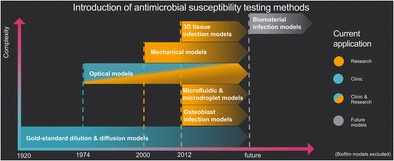
Timeline of published and partly applied antimicrobial susceptibility testing methods, listed according to their underlying mechanism, complexity and application in research or clinics.

Timeline of published and partly applied antimicrobial susceptibility testing methods, listed according to their underlying mechanism, complexity and application in research or clinics.

## Introduction

The risk of infection of a total joint replacement and in trauma surgery was described as early as in 1987 by Gristina [1], emphasising the likelihood of bacteria to adhere to implanted biomaterials, eventually resulting in infections. Yet, decades later, infections remain a complex and consequential problem for patients and surgeons. With a growing ageing population worldwide, the number of total joint replacements has also increased, leading to a concomitant increase in infection incidence. Additionally, treatment is getting increasingly difficult due to developing bacterial resistances. Periprosthetic infections, if not properly treated, eventually become chronic and engender osteomyelitis [2–4]. Different types of bacteria can trigger an infection, including those from the Enterobacteriaceae, *Pseudomonas*, *Enterococcus* and *Streptococcus* genera. Once introduced in the surrounding tissue during trauma injury or surgery, the bacteria can migrate towards the bloodstream and cause temporary bacteraemia, as well as towards foreign material (i.e. implants) to form a biofilm on the implant surface. *Staphylococcus aureus*, in combination with other bacteria such as *Staphylococcus epidermis*, has been found to be present and to trigger osteomyelitis in the majority of implant infections [2]. These bacteria not only form colonies on the implant surface, but also anchor within the host bone matrix and invade host osteoblasts [3]. A large percentage (25%) of *S. aureus* is able to undergo a change in phenotype to produce small colony variants (SCVs) [4]. These SCVs express more adhesins to facilitate uptake into non-phagocytic host cells, such as osteoblasts. The SCVs then persist in host cells by reducing their toxicity, reducing immune response to keep the host cells alive [4, 5]. Subsequently, they can reverse, exit the host cells and induce recurrent, chronic infection by becoming highly virulent again and infecting new host cells [4–8]. Bacteria are categorised by their respiratory pathway: anaerobic (survival and replication in oxygen-deprived environments), aerobic (survival and replication in oxygen-rich environments) and facultative anaerobic (bacteria, such as *S. aureus*, can grow in the presence and absence of oxygen) [9–11].

First introduced in 1929, in vitro antimicrobial susceptibility testing (AST) methods are still considered to be the most valuable in determining the efficacy of antibiotics or antimicrobial compounds against various microorganisms [12]. In general, in vitro AST methods combine one or more antimicrobial agents or materials with bacteria to assess bacterial growth. The efficacy of an agent to kill the bacteria over time can be measured in various ways, which has previously been discussed in the review of van Belkum and Dunne [13]. However, this review does not provide detailed information on the functional principle and validity of these methods. Moreover, since then, research has been published towards models that attempt to resemble in vivo functionalities. The current review, therefore, provides researchers and clinicians with an updated and more detailed insight into in vitro and in vivo-like AST possibilities to help them choose the best method for their own laboratories and research. It gives an overview of the different types of AST methods that are currently being used and discusses promising novel model developments, in particular those to study bacterial infections in osteoblasts and bone tissue. We outline the overall capabilities and efficacies of the tests, as well as their advantages and disadvantages. Furthermore, details about the ease of performance, cost, automation and commercial availability of different AST methods are provided, which should enable clinicians and researchers to choose the optimal model for specific applications (see Table 1 in the Appendix).

In this review, various AST methods are categorised based on their chronological development: 1. current gold-standard clinical methods, 2. mechanical methods, 3. optical methods, 4. microfluidics and microdroplets methods, 5. models of in vivo infection. Studies that implement bacterial biofilms in their models are excluded from this review, as in vivo-like biofilm models are much more complex and require different experimental setups and time scales.

## Gold-standard clinical antimicrobial susceptibility testing (AST) methods

Simple clinical AST methods were developed and standardised between 1920 and 1980. The gold-standard methods focus on the determination of the minimum inhibitory concentration (MIC) or the qualitative growth curve using a simple readout method for which the unaided eye is generally sufficient. Gold-standard methods are standardised by various organisations, such as the Clinical and Laboratory Standards Institute (CLSI) and the International Organization for Standardization (ISO) [12, 14–16]. The interpretation of test results is, among others, standardised by the European Committee on Antimicrobial Susceptibility Testing (EUCAST) [17]. Although the tests are reliable, they require extensive manual laboratory work and the results are normally not obtained within the same day, limiting their application. Furthermore, these tests do not offer insights about mechanisms of action.

### Agar dilution method

The agar dilution technique and the broth dilution technique had their debut in 1929. Their final versions were introduced in the 1940s and are still being used today [16]. The agar dilution method is used for the determination of the MIC of antimicrobial compounds [18, 19]. Following standardised guidelines, anaerobic [20] or aerobic [21] bacteria are seeded on nutrient agar medium, which is supplemented with different concentrations of antimicrobial agents. The colony forming units (CFU) are then counted after 48 h of incubation. This method has a simple and cost-effective readout and was found to be reliable in determining MICs [19]. However, the culture time to ensure CFU formation is long. An upgraded version is the chromogenic agar medium, which enables faster CFU detection due to an early visible colour response within 18–24 h for susceptible strains. Furthermore, it is suitable for cultures containing multiple bacteria types, as it is possible to stain only resistant bacteria, and differentiate between strains and species. The readout remains the same, but only the CFU appear coloured [22, 23]. The benefits of the agar dilution method are its simplicity and well-understood parameters. More recent AST methods, described in this review, provide greater possibilities.

### Broth macrodilution method

One of the oldest AST methods, also known as the tube dilution method, makes use of tubes containing two-fold serial dilutions of antibiotic agents in liquid growth medium [15, 24]. Subsequently, a known quantity of suspended bacteria is added to each solution. After 24 h of incubation, the bacterial growth is measured by the turbidity within the tubes, which gives an MIC value. The primary advantage of this method is the ability to obtain quantitative MIC values [19], as well as the minimum bactericidal concentration (MBC), which is the lowest amount of drug at which 99.9% of the bacteria is killed (bactericidal endpoint). This method is standardised by the CLSI for aerobically growing bacteria [25]. Standardised modifications of the method for fastidious organisms are provided in the same document.

Another analytical technique for broth macrodilution is the time–kill methodology [26, 27]. The inoculum can be multiplied in order to analyse the rate of bacterial killing for different concentrations. Viability is determined by colony counting on agar plates at regular time points over 24 h. Bacterial activity is then defined as a concentration reduction by time of ≥ 3 log_10_ CFU/mL compared to the initial inoculum. It is a standardised method described by the CLSI [25]. Time–kill curves can be drawn to visualise the antimicrobial activity of an agent, by plotting time or concentration versus the CFU/mL. By providing dynamic information between drug and bacteria, the time–kill method is especially useful for determining the bactericidal effect.

Broth macrodilution is a reliable and standardised method for diagnostic purposes. A major disadvantage is that each antibiotic solution has to be prepared by hand. Although this provides a certain degree of flexibility in drug choice and concentration, it is a very time-consuming process, especially for larger experimental investigations. Faster methods that are more suitable for screening have now replaced broth macrodilution. Additionally, a considerable amount of reagents is needed for each and every dilution. Due to the latter reason, microdilution, a downscaled version of macrodilution, has been developed.

### Disc diffusion/Kirby–Bauer method

In the same decade as the agar dilution method, diffusion techniques were discovered, which led to the introduction of the Kirby–Bauer method in 1959. Approved since 1975, this method makes use of a paper disc impregnated with antibiotics to determine drug resistance [16, 27]. Agar plates are enriched with bacterial inoculum and incubated for 18–24 h. These colonies are then resuspended in broth or saline to a standardised concentration and applied with a cotton swab on an agar plate. Subsequently, the paper discs are placed and the plates are incubated overnight. An inhibitory zone arises where the concentration of the diffused antibiotic particles is high enough to inhibit bacterial growth. Qualitative results are provided, categorising bacteria as susceptible, intermediate and resistant strains, depending on the size of the inhibitory zone. Standardised readouts are available for the assessment of the inhibitory zone of many bacteria types and strains, as well as for drug interactions by introducing multiple discs containing different drugs on the same agar plate [28]. Disc diffusion is standardised for rapidly growing aerobic pathogens as well as several fastidious organisms, such as *Haemophilus influenzae*, *Neisseria gonorrhoeae*, *N. meningitidis*, *S. pneumoniae*, and beta-haemolytic and viridans group streptococci [29]. Modifications of the method may enable the testing of other fastidious bacteria as well [30]. The handling of the test is simple, straightforward and does not require any special equipment. The possibility to change the type of antibiotic by simply changing a single disc provides a certain degree of added flexibility. Currently, this AST method has the lowest costs [15]. A significant disadvantage is its unsuitability for slow and anaerobically growing microorganisms [27, 29]. Furthermore, this test is dependent on appropriate diffusion. As such, the molecular weight of the drug molecules is a crucial factor when using this method. In addition, imperfections and unevenness of the agar plates can affect diffusion and lead to false results. Another drawback is that the test only provides qualitative results and no quantitative MIC values [15]. More complex approaches, as described in the following paragraphs, have attempted to address some of these disadvantages.

### Etest

**Fig. 1:**
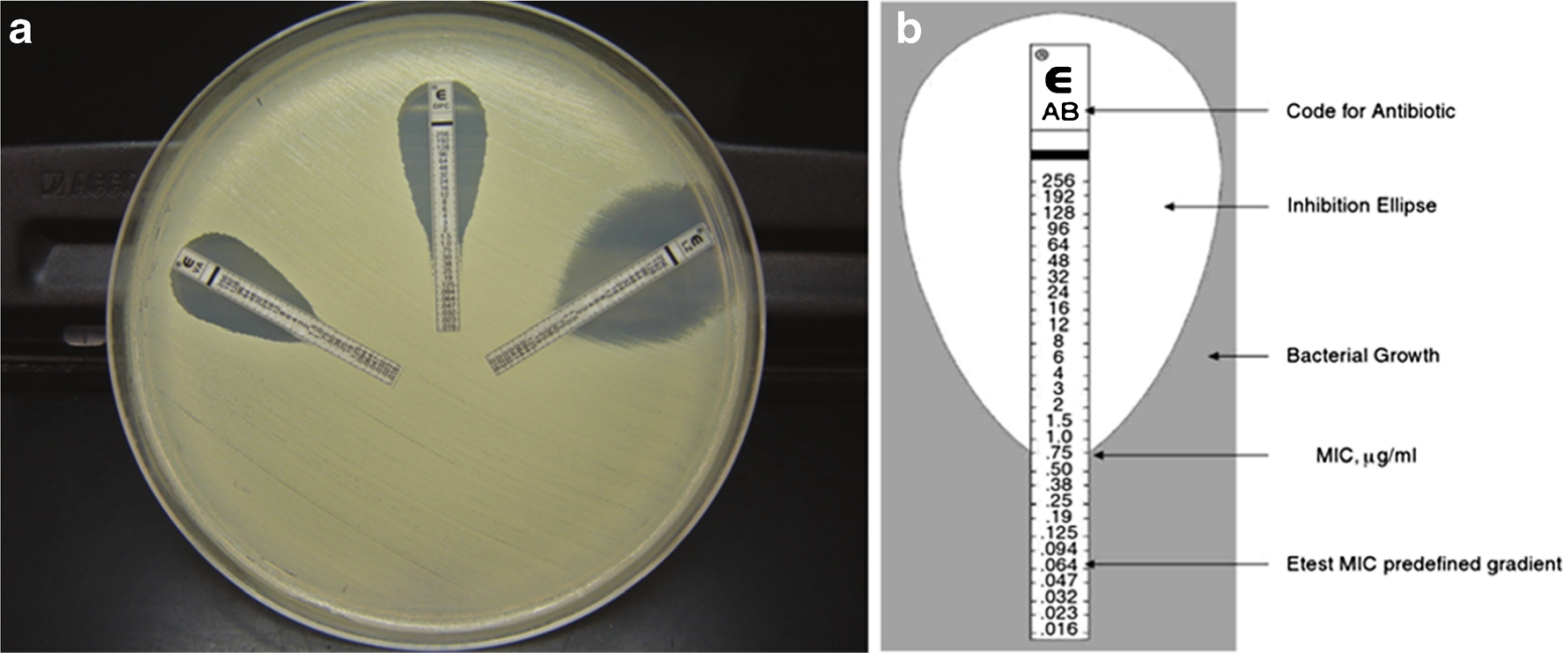
Etest for determining the minimum inhibitory concentration (MIC) of antibiotics. **a** Image of the test setup. The applied antibiotic concentration on the strip depends on the type of antibiotic and can vary between 0.016–256 μg/mL or 0.002–32 μg/mL. **b** Schematic representation of the Etest inhibition zone, indicating the MIC at 0.75 μm/mL. **a** Adapted from Jorgensen and Ferraro [15] with permission from Oxford University Press. **b** Adapted from Schwalbe et al. [27] and Taylor and Francis Group LLC Books

The Etest is an extended version of the agar diffusion method and performed similarly to the disc diffusion method. Instead of the paper discs, a predefined exponential gradient of antibiotic agent is applied to the bottom of a plastic strip, which is subsequently placed on an agar-based medium to generate diffusion of the drug (Fig. 1). After 24 h of incubation with the bacterial inoculum, the exact MIC of a drug that is necessary to stop bacterial growth is easily read on the strip.

Another technique with comparable functionality is the spiral gradient endpoint technique, where the gradient is produced by a spiral plater that deposits a stock solution on the agar plate. The distance from the centre of the plate to the endpoint of growth enables the determination of the MIC [31], as previously reviewed by Jorgensen and Ferraro [15]. This method was found to be an effective method for the detection of heterogeneous vancomycin-non-susceptible *S. aureus* (hVISA) and vancomycin-intermediate *S. aureus* (VISA) [31].

The Etest has several advantages over the disc diffusion test. First, because the gradient is stable for up to 20 h, it is suitable for a variety of pathogens, ranging from rapid-growing aerobic and anaerobic bacteria to slow-growing fastidious bacteria [32]. In addition, the test can be used for finding heterogeneous resistance, or a bacterial strain that forms both susceptible and resistant colonies [31]. It can provide an exact MIC value, which allows fine-tuning of the antibiotic treatment regimens of patients. However, this method is more expensive than the paper disc diffusion method [15, 27]. Jorgensen and Ferraro [15, 33] also found a systematic bias for smaller and larger MIC values for specific bacteria–drug combinations compared to broth dilution, demonstrating a limitation of the test. In general, the Etest is a convenient AST method that provides exact quantitative MICs at a higher cost.

### Microdilution method

Microdilution was pioneered in 1977. Microdilution attempts to increase the throughput of broth macrodilution by conducting one test using 12 different antibiotic agents in a 96-well plate. After the manual or automated addition of a small amount of bacteria to each well, the plate is incubated overnight, producing an observable colour change, along with bacterial growth. An automatic or manual viewing device can be used to determine the MIC by fluorescence intensity or turbidity measurements [15]. The well plates can be purchased containing different concentrations of the dry agent pre-prepared in each well, improving standardisation and ease of use. Microdilution is standardised by the CLSI for both anaerobic [34] and aerobic bacteria [21]. The most obvious benefit is the reduction in workspace, reagents and time. However, a major drawback is that drug selection is limited to commercially available panels. With a higher degree of automation, microdilution is now a gold-standard technique preferred over broth macrodilution.

### Microcalorimetry

**Fig. 2:**
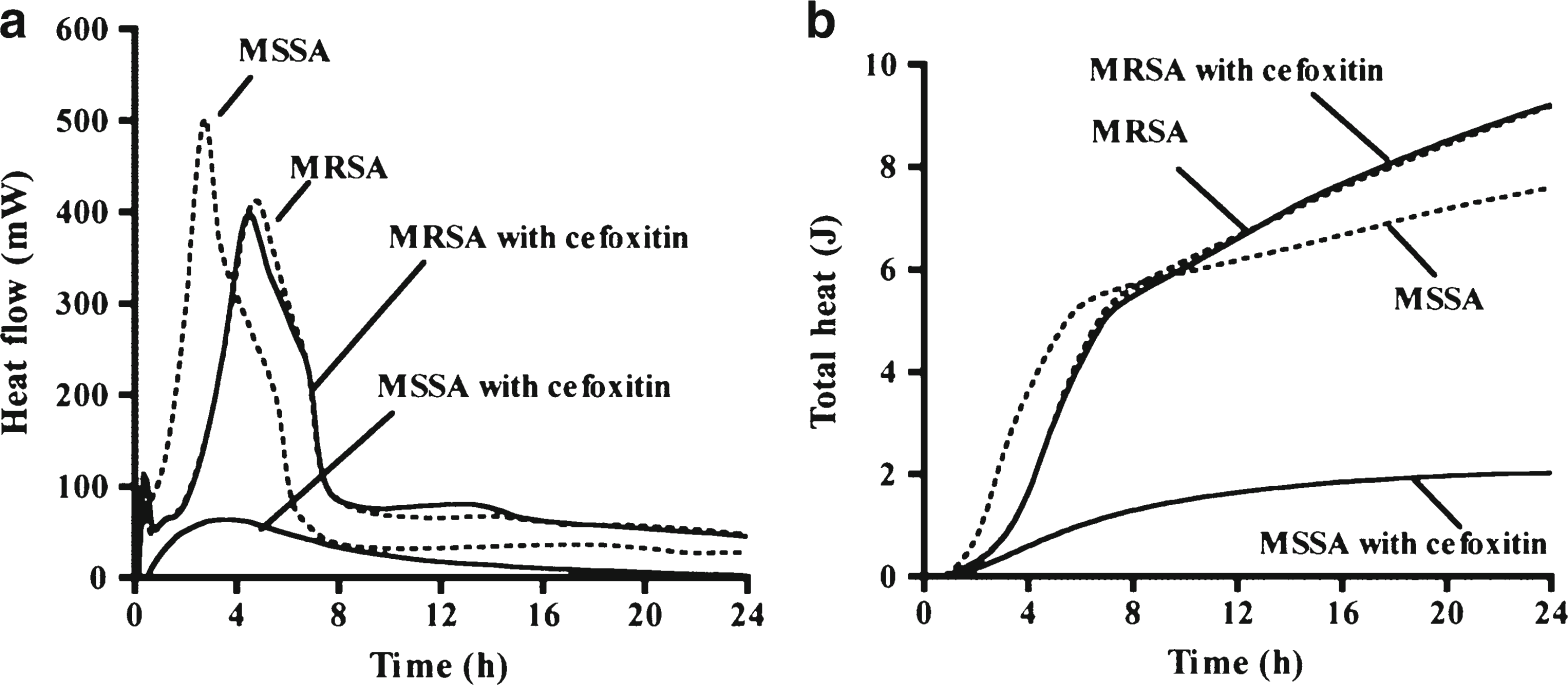
Microcalorimetry shows **a** heat flow (mW) and **b** total heat (J) generated by microbial growth over time (h). Differences can be detected between methicillin-sensitive *Staphylococcus aureus* (MSSA) and methicillin-resistant *S. aureus* (MRSA) in the presence (*solid lines*) and absence (*dashed lines*) of cefoxitin. Adapted from Baldoni et al. [38] with permission from the American Society for Microbiology (ASM) Journals

Microcalorimetry utilises the thermal events of bacterial growth to detect bacterial susceptibility. The test pioneered as an alternative AST method in the mid-1970s [35, 36]. Several studies have been conducted in the past decade using a microcalorimetric assay to determine the MICs of various *S. aureus* and *Escherichia coli* strains [37], and to detect methicillin-resistant *S. aureus* (MRSA) [38, 39]. During the exponential growth phase of bacteria, an exponential increase in heat production takes place due to expanded cellular metabolism. This heat flow can be measured by a calorimeter, and plotting the heat pattern over time allows the generation of a bacterial growth curve (Fig. 2). MRSA and methicillin-sensitive *S. aureus* (MSSA) were shown to exhibit different heat development profiles after 5 h and after 4 h by Baldoni et al. [38] and von Ah et al. [39], respectively. The possibility to differentiate antibiotic-susceptible and -resistant bacterial strains in such a short time is a major advantage and makes it an interesting tool for screening. Microcalorimetry is an informative method, which provides MIC values, the general rate and curve of bacterial growth, antibiotic susceptibility and resistance. Even though microcalorimetric results were found to be in line with standardised methods [40], there are no official documents of standardisation available. Additionally, the amount of research done in measuring heat production for an AST method is limited.

### Polymerase chain reaction (PCR)-based techniques

Polymerase chain reaction (PCR)-based techniques for AST are commercially available in the form of assays and automated machines, and are highly favoured in clinical settings. PCR, both real-time and conventional, are used to amplify DNA sequences that are specific to a particular pathogen and its susceptibility or resistance to a drug. An example includes the *mec*A gene that encodes the penicillin-binding protein PBP2a, which reduces the affinity for beta-lactam antibiotics [41, 42]. This gene is commonly found in MRSA and it is highly advantageous in that it can be quickly identified in clinics [43]. Furthermore, the extent and intensity of gene expression and not just its presence are important parameters, as some genes need a high expression in order to produce resistance [44].

One major advantage of PCR-based methods is that they can be conducted and evaluated rapidly. Results can be obtained with high specificity and sensitivity within 2 h. Furthermore, samples do not always have to be purified, but can be primary, non-sterile, clinical samples, and, thus, can contain bacterial mixtures [45–48]. Real-time PCR can detect differences between susceptible and resistant strains with very short incubation time and has been applied for a variety of bacterial types, particularly MRSA and vancomycin-resistant MRSA (VMRSA) [49–53]. It can also distinguish between live and dead bacteria by using, for example, ethidium monoazide or propidium monoazide [54, 55]. On the other hand, PCR techniques cannot provide information about mechanisms of resistance [42]. Furthermore, the number of genes and mechanisms involved in resistance can be quite complex depending on the strain, which can lead to misinterpretation of data [56–58]. Knowing which genes to analyse is a prerequisite to minimise the possibility of detecting false resistance, and this remains one of the major drawbacks of this method. Nevertheless, PCR-based AST methods are a safe, efficient and reliable screening tool in clinical settings.

## Mechanical methods

Since the turn of the century, mechanical methods have been developed for AST. To date, they primarily serve in research applications and are not affiliated with clinical diagnostics. Magnets, electrodes or cantilevers are introduced in the bacterial culture to measure variables such as rotational and resonance frequency, as well as impedance to assess bacterial growth, even for small amounts of bacteria. Their setup is usually complex and the readout is microscope- or computer-aided and, therefore, largely automated.

### Asynchronous magnetic bead rotation sensor

**Fig. 3:**
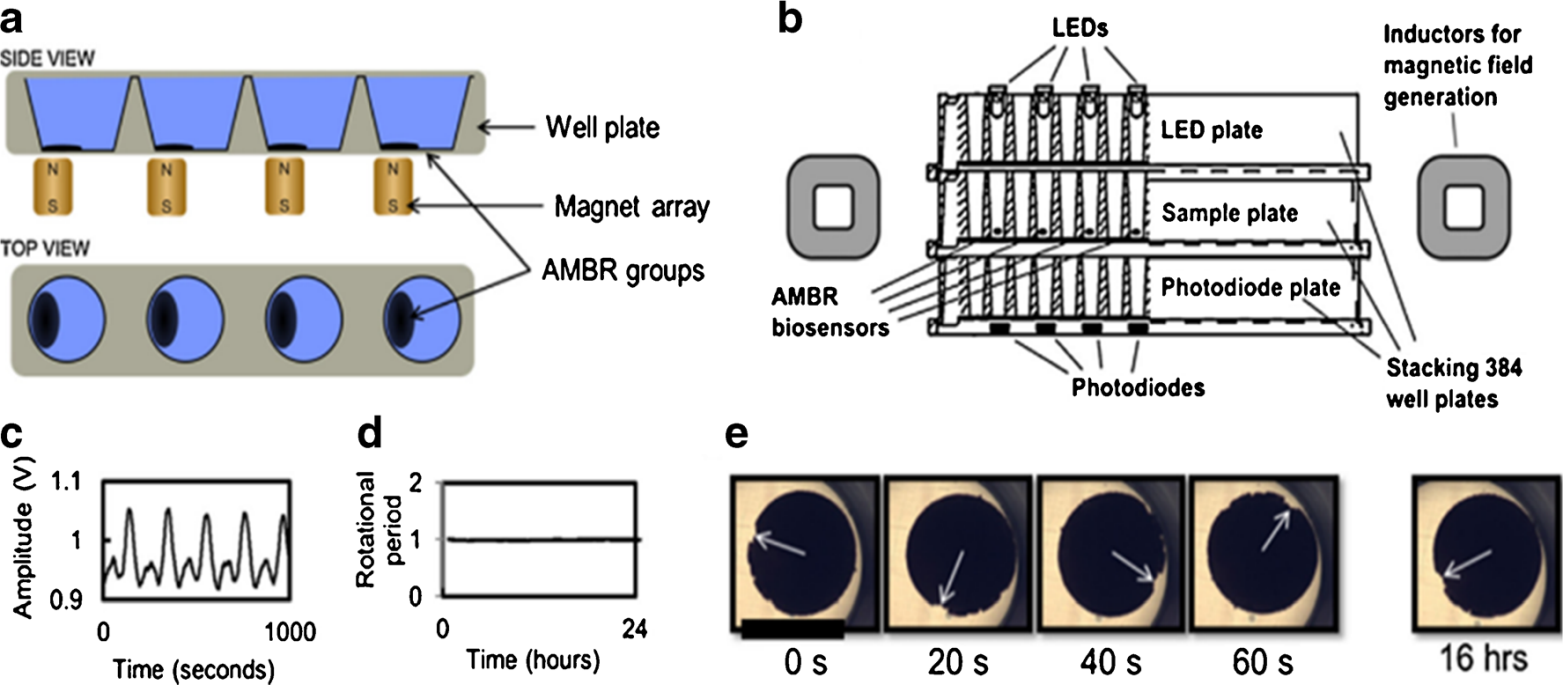
Asynchronous magnetic bead rotation (AMBR) sensor consisting of self-assembled magnetic particles to which bacteria adhere. Applying a magnetic field results in rotation of the magnetic beads. Bacterial growth leads to a change in viscosity and, subsequently, in the amount of rotational periods. **a** Side and top views of the array showing sample and sensor. **b** Cross-sectional view of the setup. **c** Data of a single photo diode, with every peak representing one rotation of the sensor. **d** Normalisation of the rotational period over 24 h. **e** Microscopic images of the AMBR sensor rotation in one well at different time points. Adapted from Kinnunen et al. [59] with permission from Elsevier

As a novel method in its proof-of-principle stage, the asynchronous magnetic bead rotation (AMBR) sensor is used to determine bacterial growth by measuring the viscosity of a bacterial solution. In this method, bacterial cells at a known concentration are mixed with magnetic particles with a diameter of 2.8 μm and incubated under rotation for 10 min at 37 °C [59]. After a washing step, the solution is pipetted into a well plate and placed on top of a permanent magnet array for 5 min, in order to induce magnetic self-assembly of the particles into AMBR sensors. Each AMBR sensor consists of approximately 10^6^ magnetic beads. As shown in Fig. 3, the rotational periods of the AMBR are measured by photodiodes through the bottom of the well plate by measuring the fluctuating light. The amount of rotational periods of the AMBR sensor decreases when bacteria, which are adhered to the sensor, start to grow. Upon reaching a certain number, the accumulated bacteria start to expand and, therefore, the amount of rotational periods increases again. The AMBR method has the advantage that the initial growth of small concentrations of bacteria can be assessed within approximately 1.5 h; this rapid detection makes it useful for screening. However, the setup is complex and might not be applicable in the clinic. Because AMBR has only been applied to *E. coli*, it remains to be determined whether it is capable of identifying resistant strains. More research is required to determine its sensitivity, specificity and final application. If these results turn out to be promising, an automated version would make clinical application interesting. Sinn et al. [60] built a more isolated setup, using the same mechanisms. Their AMBR microviscometer platform involved microfluidic droplets, in which bacterial growth was assessed by determining the viscosity of the droplets. Determination of the MIC, however, takes 6–24 h and it has been tested for *E. coli* only. Speeding up the process and applying it to different bacteria would make it an interesting application.

### Single-cell AMBR biosensor

**Fig. 4:**
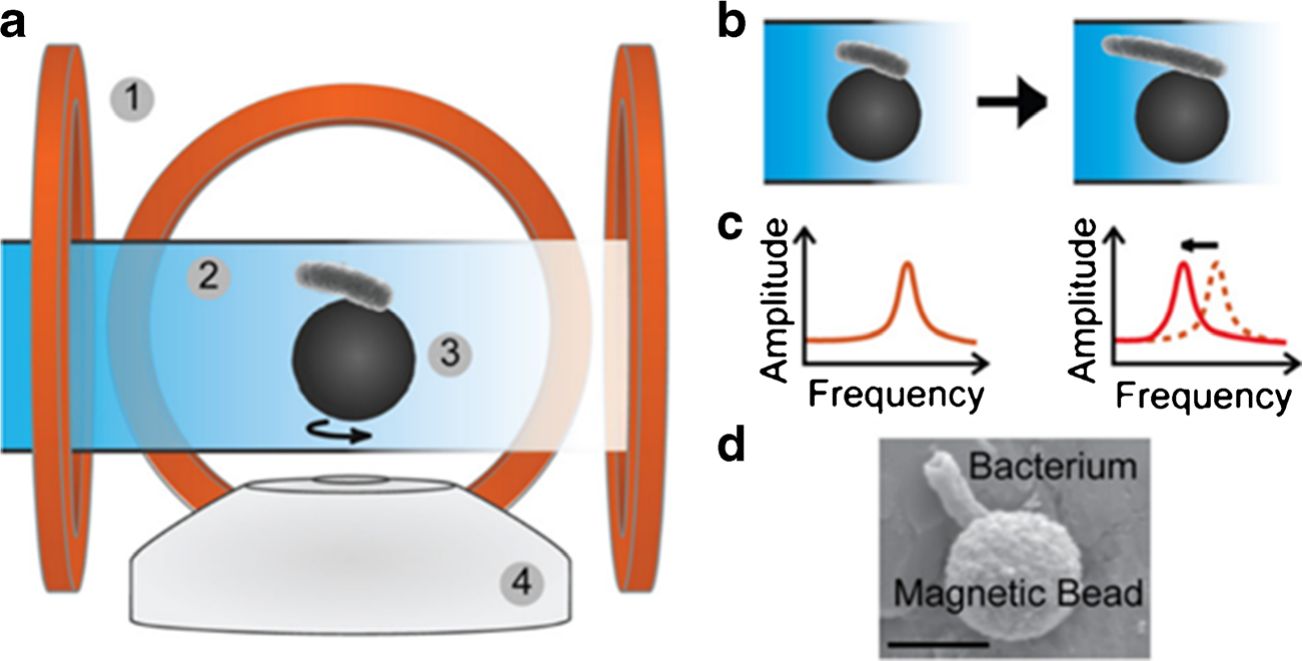
Single-cell analysis of *Escherichia coli* with an AMBR biosensor. **a** Schematic setup of the method: electromagnetic coils (*1*) surround a single bacterium (*2*) that is attached to a magnetic bead (*3*). **b** A single cell is attached to a magnetic bead whose rotation is induced by a magnetic field. **c** Frequency and amplitude changes represent changes in bacterial growth. **d** Micrograph of a single *E. coli* bacterium attached to a magnetic bead. The scale bar represents 2 μm. Adapted from Kinnunen et al. [61] with permission from Elsevier

In another version of the AMBR sensor, developed by Kinnunen et al. [61], a single ball-shaped magnet is used instead of self-assembled biosensors, to which a single *E. coli* bacterium can be attached (Fig. 4). Using this technique, cell growth throughout the whole life cycle and cell division of a single cell can be visualised and analysed microscopically under the influence of antimicrobial compounds. Although research with this method is still limited to *E. coli*, yeast and cancer cells, it shows potential for testing various bacteria and drugs. However, it is more applicable to research than clinical settings, due to its small scale and complex setup. Still in its proof-of-principle stage, this method still requires much validation. Theoretically, it could be combined with a microfluidics method in order to analyse additional factors such as temperature, pH, nutrients and mineral levels by creating a bioreactor-like environment.

### Surface acoustic wave sensor

The surface acoustic wave (SAW) sensor is a method to determine the amount of cells in a culture by placing electrodes in the culture solution, which measure frequency changes, caused by an impedance change of microbial metabolism. Conductive electrodes are placed in the culture solution of interest and the time is measured until frequency changes are detected [62]. To detect *E. coli* growth from an initial solution of 100 cells/mL, only a 7-h measurement was required. This is faster than most gold-standard AST methods. No pre-culture is required, as long as the starting amount of cells is very low. As an additional advantage, the system operates wirelessly and, therefore, offers the opportunity for isolated, safe bacterial work with highly virulent strains. To date, SAW has been applied only to *E. coli*; therefore, testing and validation with other bacteria are necessary to determine its potential applications. Unfortunately, measurements are prone to building up noise and are sensitive to frequency changes caused by protein adhesion to electrodes, temperature changes and the presence of amino acids with acidic or basic residues. Thus, the pH value of the assay must remain constant [62, 63], which makes longer bacterial culture or even co-culture difficult to pursue. The SAW method is used for cells in suspension, which means that clinical samples would need a pre-treatment in order to be analysed. Even though a clear number of cells/mL can be determined, this method does not provide data about resistance and mechanism(s) of resistance [62, 63].

### Microbial cell weighting with vibrating cantilevers

Another quantitative method involves vibrating cantilevers that make use of the buoyant mass principle [64]. A bacterial suspension flows through a channel within a resonating cantilever. The position of bacterial cells on the cantilever changes the total cantilever mass. This leads to a change in resonance frequency, which can be directly related to the buoyant mass of the cell. The cellular, buoyant density usually differs per cell species. As this method allows single-cell analysis of volume, mass and density, a precise growth rate can be determined as well as bacterial adhesion to cells [64–66]. Vibrating cantilevers have already been used in antimicrobial susceptibility research and various other fields. In addition to cancer cells, viruses, and mouse and human T cells, research using this method has been performed for yeast, *E. coli*, *E. faecalis* and *Citrobacter rodentium* [67–72]. One benefit is that the overall growth rate and cell type can be determined with a notably small amount of cells. More research is necessary, however, for the application of this method for AST purposes to ensure reliable detection of various resistant strains and bacteria types. Furthermore, this is a rapid method, with results obtainable in 20 min to 2 h, as no pre-culture is required [70, 72]. Microchannel cantilevers also have the potential of high-throughput measurements and are, therefore, suitable for screening [73]. It still needs to be validated if cultures of multiple bacteria and co-cultures with human cells are possible.

## Optical methods

Since their introduction in the mid-1970s, optical methods have developed into sophisticated tools over the years. Generally, light beams are used to detect, stimulate or trap bacterial cells. Common variables measured are bacterial motion, molecular vibrations, bacterial density and fluorescence intensity. Depending on the method, bacterial strains can be identified and a bacterial growth curve as well as quantitative MICs can be determined. Because they rely on expensive and complex spectroscopic readout methods, optical methods are mostly applied in research settings.

### Optical density (OD) measurement

**Fig. 5:**
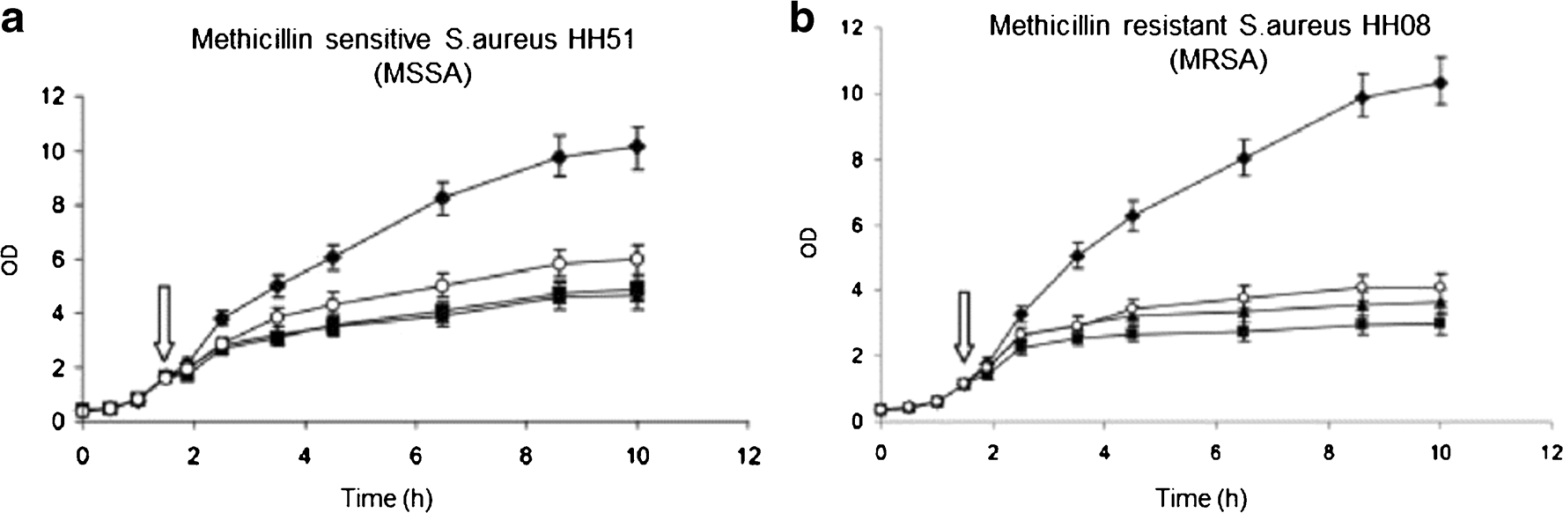
Bacterial growth curve of methicillin-sensitive *S. aureus* (MSSA) **a** and methicillin-resistant *S. aureus* (MRSA) **b** strains. Optical density (OD) is determined over time. The *filled diamonds* indicate untreated strains, while the other shapes represent three different kinds of antibiotic treatments. The *arrows* indicate OD ~ 1. Adapted from Kilian et al. [74] with permission from John Wiley and Sons

Optical density (OD) measurements with a spectrophotometer can determine the growth curve of bacteria over time in a liquid sample [74–78]. Initial attempts to measure the bacterial growth by OD were performed in 1974 [79], but found greater application after the turn of the century. The OD of a bacterial suspension is determined at regular time points upon exposure to an antimicrobial compound, and can, thus, discriminate resistant and sensitive strains (Fig. 5). An OD_600_ of 1 conforms to the exponential growth phase of the bacteria (see the arrows in Fig. 5). OD measurements have been done for various *E. coli* and *S. aureus* strains, but also *S. epidermis*, *S. enterica* Newport and *E. faecalis* [80]. More research is needed to validate the method for a greater variety of bacteria. Although there is no official standardised protocol for OD measurements, protocols for bacterial suspensions are available from the CLSI, as previously shown for the dilution methods. The main advantage of this method is the fast indication for growth, determining the exponential phase within a couple of hours. It is also non-destructive and inexpensive, which makes it an interesting screening tool. However, the determination of bacterial growth is only an estimation of CFU in solution and cannot determine MICs. The method is not suitable for very low concentrations of bacteria, and it is likely to become unreliable when bacteria are cultured with a dissolving substrate that could interfere. Additionally, co-culture and infection studies with human adherent cells that normally proliferate only when attached to surfaces are not possible, as the bacteria need to stay in solution.

### Biomimetic polymer sensor

The biomimetic polymer sensor is a fast, automated assay based on the principles of agar plate CFU counting [81]. Membrane-active compounds such as peptides and toxins that are secreted by bacteria interact with agar-embedded nanoparticles, made of phospholipids and chromatic polymer polydiacetylene (PDA). During interaction with these compounds, PDA undergoes a transition from blue to red and starts to emit intense fluorescence at 560 nm and 640 nm, which can be detected by common high-throughput screening instruments, such as multi-well fluorescence plate readers. The initial fluorescence signals indicating the colour change are detectable in less than 6 h, faster than the bacterial colony formation visible to the naked eye. Therefore, it is a much faster and easier method to determine bacterial growth than the common agar plate counting. In addition, it is possible to incorporate antimicrobial agents into the chromatic matrix, which enables the simultaneous detection of antibiotic susceptibility while monitoring bacterial growth. Such a measurement can also be performed at the single-cell level, as a single cell can be fluorescently detected. This method has been proven suitable for various *E. coli* strains, *Bacillus cereus* and *S. enterica*, and is potentially applicable to a wide range of bacteria, including Gram-positive and -negative strains. One major disadvantage of this technique is that it does not differentiate between bacterial species and is not suitable for screening unknown pathogens. Bacterial mixtures, common for clinical samples, cannot be analysed by this method. Within the study of Silbert et al. [81], the variability of colour responses between experiments was reported to be 10–15%, which may limit the suitability of the method for precise MIC determination. Furthermore, no official standardised protocol is available. Despite these disadvantages that require more research, the PDA sensor method is a fast screening alternative to the gold-standard agar plate counting method.

### Flow cytometry and fluorescence-activated cell sorting (FACS)

Fluorescence-activated cell sorting (FACS), a method of flow cytometry, is used to distinguish between cell types and to determine their viability and count by light scattering and fluorescent activity. Bacterial viability and antibiotic susceptibility can be determined by combining bacteria, antibiotics and relevant fluorescent stains [82]. After 2 h of incubation of cells with fluorescent stains, the sample can be analysed by flow cytometry. Standardised protocols and standardised data interpretation are not available for AST and vary for each pathogen and antimicrobial agent. So far, flow cytometry in AST has primarily been used for MRSA [83, 84], MSSA [83, 84], *E. coli* [82] and *S. enterica* [85]. As certain fluorescent stains can bind to nucleic acids within the bacterial cells, permeabilised cells give stronger fluorescent emission than non-permeabilised cells, and completely lysed cells give an even greater amount of fluorescence. For example, MRSA were found to show a greater population of viable cells than MSSA after incubation with oxacillin [83]. However, the amount of fluorescence activity is not necessarily in direct correlation with the viability of the cells, due to the fact that flow cytometry inadequately distinguishes single cells from cell aggregates. Second, the amount of fluorescence is dependent on size and metabolic characteristics that vary per bacterial strain, and are also dependent on the antibiotic used. Third, some antibiotics can alter the extent of permeability of bacteria by inducing a membrane potential [83]. Cell lysis often occurs due to exposure to β-lactams and results in brighter fluorescent emission without a change in the number of cells [86]. Flow cytometric results and images taken with an epifluorescence microscope should, therefore, be interpreted carefully. Shrestha et al. [83] as well as Sánchez-Romero and Casadesús [85] found that antibiotic resistance could be determined and distinguished from susceptible strains after 2 h with both side and forward scatter using flow cytometry. Another benefit of this technique is that it can be used for prokaryotic and eukaryotic cells. Within a co-culture of bacteria and osteoblasts to study infection, both cell types can be distinguished and analysed by their viability over time, even in the presence of antimicrobial compounds. With its fast performance and wide potential, flow cytometry could find powerful application in research and in clinical screening. However, extensive research is needed to improve its validity.

### Image analysis software for quantification of bacterial growth

Bacterial growth can also be analysed by taking images of different growth stages and quantifying them with an image analysis program. There are different types of software available, such as CellProfiler [87], Colony Imager [88], YeastXtract [89] and HT Colony Analyser [90]. Colonyzer, for example, is a software program that automatically quantifies the size, granularity, colour and location of microbial organisms on solid agar plates [88]. It includes a collection of image analysis algorithms that can detect low-density organisms from plate micrographs that are barely visible to the human eye. Most software programs for quantifications are open source, and when using only agar colonisation, this method is relatively cheap, with easy handling and is applicable for a variety of pathogens. So-called digital plate reading systems are available and already thoroughly discussed [91]. Due to their high sensitivity, image analysis software and digital plate reading systems are able to detect early growth of pathogens. MICs can, therefore, be determined much faster. Furthermore, the exposure to pathogens is decreased when closed systems are used and common laboratory work, such as plate labelling, is not necessary due to automation. Some disadvantages of image analysis software or digital plate reading systems are that co-culture with eukaryotic cells is not possible with agar plates, and antimicrobial release from a substrate other than solid agar is not available. In order to determine the exact MIC, many consecutive images have to be taken and analysed, which can still be time-consuming, depending on the software, amount of samples and extent of automation. In summary, the image analysis software available is a reliable method for AST under appropriate conditions.

### Optical tweezers

In a proof-of-principle study, Samadi et al. [92] showed that a single bacterium can be trapped in an optical tweezer, a 1064-nm laser beam, in order to assess the effect of chemical agents. The highly diluted sample is mounted on a piezoelectric transducer. The light scattered forward from the trapped cell is collected and sent to a position-sensitive detector. With statistical analysis of the position signal at different time points, the viability can be determined. This measurement is sensitive to such an extent that flagellar motion can be distinguished from the Brownian motion. Depending on the concentration of the chemical agent and bacteria type, the single-cell response can be determined within a couple of minutes, which is a major advantage. The single-cell trapping makes it a safe application for research and offers the opportunity for in-depth analysis of bacterial viability and the precise “killing time” upon antimicrobial exposure. Precise MICs can be determined without growing colonies or pre-culturing the cells. However, the system is only applicable for motile bacteria. For AST applications, this method has been demonstrated only for *E. coli* and, therefore, has no standardised protocols. With further research and validation, this technique could be promising due to its high-sensitivity capabilities.

### Raman spectroscopy

Raman spectroscopy has also been demonstrated to be a precise technique to measure the susceptibility of bacteria to antimicrobial compounds [93–95]. Using a frequency-doubled argon ion laser at an excitation wavelength of 244 nm, Raman scattering of aromatic amino acids and nucleic acid bases is induced within bacterial cells and can be detected by vibrational bands. The total cell mass can be determined over time and plotted, giving the growth curve for the different bacterial growth phases, starting with the log phase, followed by the exponential growth phase, retardation phase and stationary phase prior to cell death. Every single phase can be seen within Raman spectra. Common analysis techniques include hierarchical cluster analysis (HCA) and principal component analysis (PCA). Raman spectroscopy can provide information about the underlying mechanism by which a drug affects the bacterial cells with the help of statistical methods. A different Raman technique, called the laser tweezers Raman spectroscopy (LTRS), allows single-cell analysis. In contrast to the common Raman technique, LTRS is applicable to low bacterial concentrations and distinguishes cellular drug response from normal growth of *E. coli* after 4.5 h [96, 97].

Raman spectroscopy has the advantage that any type of microbe and antimicrobial compound can be analysed, as long as nucleic acids and proteins are present to a sufficient extent, and size and migration characteristics are taken into account. Additionally, Raman spectroscopy is highly sensitive regarding which bands of nucleic acids and which building blocks of proteins are excited. This makes it possible to assess the actual mechanism of antimicrobial compounds to kill microbes. One disadvantage is the high costs of Raman spectroscopes, although running costs are relatively low. Even though Raman spectroscopy has been used in clinics as a diagnostic tool for cancer [98], it has not yet been applied as AST method.

### Matrix-assisted laser desorption/ionisation time-of-flight mass spectrometry (MALDI-TOF MS)

**Fig. 6:**
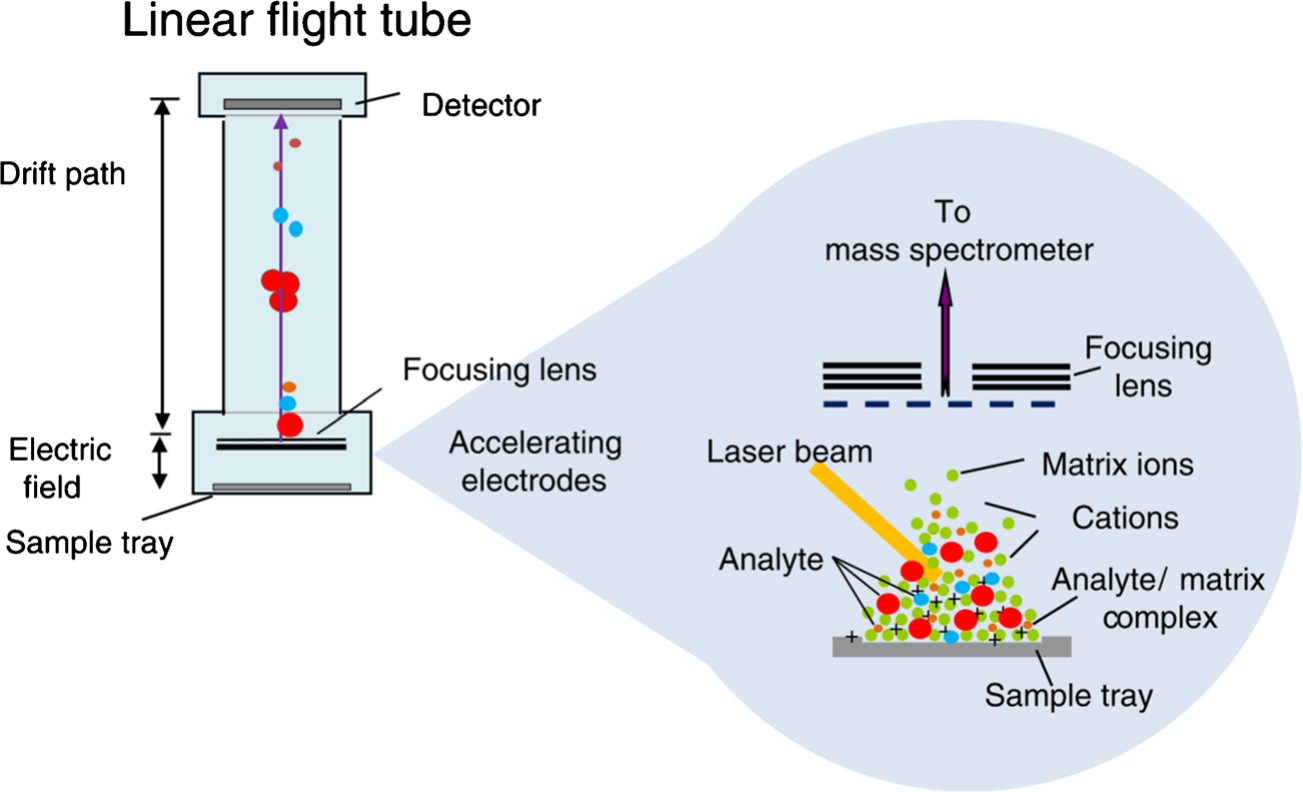
Schematic representation of matrix-assisted laser desorption/ionisation time-of-flight mass spectrometry (MALDI-TOF MS), in which soft ionisation of the microbial sample by laser pulses (*right*) results in ion displacement through the linear flight tube (*left*). Differences in flight time to the detector represent differences in ribosomal proteins. Reprinted from Wieser et al. [100] with permission from Springer

Introduced in 2005 as an AST method [99], matrix-assisted laser desorption/ionisation time-of-flight mass spectrometry (MALDI-TOF MS) is a mass spectrometric technique that uses soft ionisation by laser pulses for the analysis of biomolecules. Ions are accelerated in this process and their respective time of flight is measured, where the smallest travel the fastest (Fig. 6). Thereby, the mass-to-charge ratio of a variety of compounds is determined, and species and strains can be distinguished based on their ribosomal proteins. Analyte-specific spectra are produced that can be compared with databases for pathogen identification. This technique can distinguish between pathogens in blood and urine samples, as well as bacterial strains in a bacterial mixture within approximately 30 min. With these rapid processing times, MALDI-TOF has been deployed for AST in the clinics [100, 101]. Even though acquisition costs for the equipment are high, operating costs per analysis are very low. It has, therefore, already been introduced in clinical microbiology laboratories [102]. Furthermore, the system does not depend on metabolic reactions and can be used for Gram-positive and -negative bacteria and yeast [100, 103, 104]. MALDI-TOF MS, however, suffers from several disadvantages. Samples must be analysed within 48 h of collection, otherwise the peaks in the spectra are difficult to distinguish, plausibly due to ribosomal protein degradation. Automation of processes is advised, as most samples, except for urine, require various time-consuming preparation steps [100, 105]. Whereas the identification of pathogens is successful, strain identification to assess resistance remains an issue in the cases of limited difference in ribosomal sequences. Therefore, distinguishing resistant strains unequivocally from non-resistant ones is not possible. Indeed, results on the efficacy of MALDI-TOF to differentiate between MRSA and MSSA are contradictory [100, 105]. Overall, this relatively new method opens new possibilities for reliability and efficiency in AST, though further validation is required.

### Rapid antimicrobial susceptibility testing (RAST) methods

Beginning in the mid-1970s, research efforts have focused on improving these gold-standard AST methods, in order to reduce costs, time and effort. Rapid antimicrobial susceptibility testing (RAST) methods are optical detection systems that facilitate the observation of small changes in bacterial growth and were assessed with gold-standard methods such as Kirby–Bauer or agar dilution as the control. The Autobac 1 system [106] was introduced in 1975 as an RAST method. Other machines, such the Vitek2, BAXTER MicroScan Walkaway, BD Phoenix Automated Microbiology System and Sensititre ARIS 2X, followed and are FDA-approved for clinical application [14, 107–110]. All use similar techniques, including microdilution trays or small cards that are inoculated with a known concentration of bacteria. Subsequently, multiple antibiotic tests can be performed on the same tray/card. Readout is done by photometers or fluorometers, which monitor reactions in each well and report changes within 3 to 24 h. The underlying software is able to integrate information from multiple reactions, to identify microorganisms and to comment on mechanisms of resistance patterns. Depending on the system, it is possible to use untreated clinical samples. Various studies compared these systems with gold-standard techniques and found that automated systems accelerated patient treatment, decreased the mortality rate and were cost-saving [107, 108, 110]. Newer methods were found to deliver even more reliable results, showing that even well-established automated systems have their constraints [111]. However, a detailed discussion on the reliability and constraints of RAST methods is beyond the scope of this review. To summarise, automated systems show, due to their speed and resulting decrease in mortality rate and cost-savings, clear benefits as diagnostic tools in the clinic.

## Microfluidics and microdroplets methods

Developed in the last decade, microfluidics and microdroplets methods enable the building of complex in vitro systems on one chip. By the use of small amounts of fluids through channels in the micrometre range, chips can be designed on which several functionalities, such as bacterial culture, nucleic acid hybridisation and amplification, and cell lysis, can be combined. These hold the major advantages of “temporal and special control over fluids and physical parameters, and integration of sensors to obtain direct and in situ read-out” [112]. So far, detection within microfluidic AST methods is generally electrochemical or optical/microcalorimetric. These methods provide rapid results and show the potential for high throughput, due to a high surface-to-volume ratio. Currently, microfluidic AST methods have been used only for antimicrobial research, but hold great potential for clinical application for several reasons, such as a reduction in workload and materials [113]. Besides the following methods, interested readers are also referred to more research highlights within antimicrobial microfluidics [114].

### Microfluidic agarose channels (MAC) tracking single cell growth

**Fig. 7:**
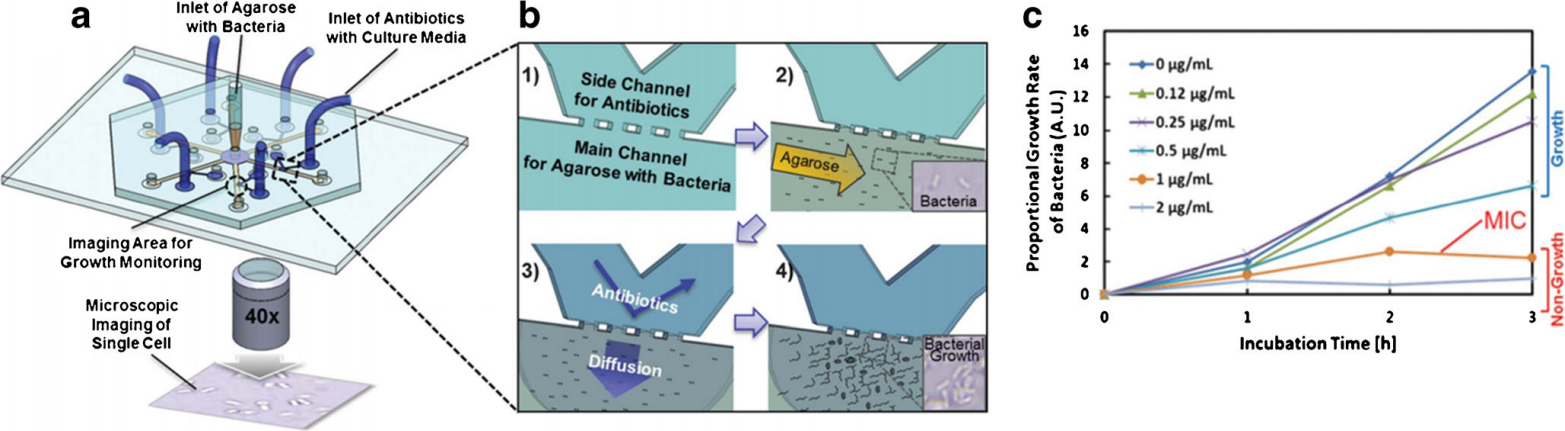
Microfluidic agarose channel (MAC) system. **a** Schematic showing the agarose–bacteria solution injected through the main inlet in the centre of the chip. Antibiotics added to cell culture medium are introduced through the six channel inlets **b** and the bacterial growth is imaged at different time points. **c** From the images, the proportional growth rate of *S. aureus* is plotted over time in order to determine the MIC. Adapted with permission from Choi et al. [115]. Copyright (2017) American Chemical Society

Microfluidic agarose channels (MAC) represent a fast RAST method for the determination of MICs by single-cell bacterial time-lapse imaging. Bacteria, *S. aureus* and *P. aeruginosa*, are immobilised on agarose in a microfluidic culture chamber and exposed to different antibiotic culture conditions, which enables the analysis of single cell growth (Fig. 7a, b). Time-lapse images are taken with a CCD camera and the proportional growth rate can be determined and plotted against the incubation time to determine MICs (Fig. 7c) [115].

This method enables single-cell analysis in a closed system, which creates a safer work environment. Data (MIC values) can be obtained within 3–4 h, which is highly advantageous compared to standard clinical AST methods for fast patient diagnosis and treatment. One disadvantage is that clinical samples need several preparation steps before the analysis can be performed. The MAC system is an improvement regarding MIC determination, compared to gold-standard methods. Whether conclusions on resistance can also be drawn still needs to be investigated.

### Microfluidic pH sensor

**Fig. 8:**
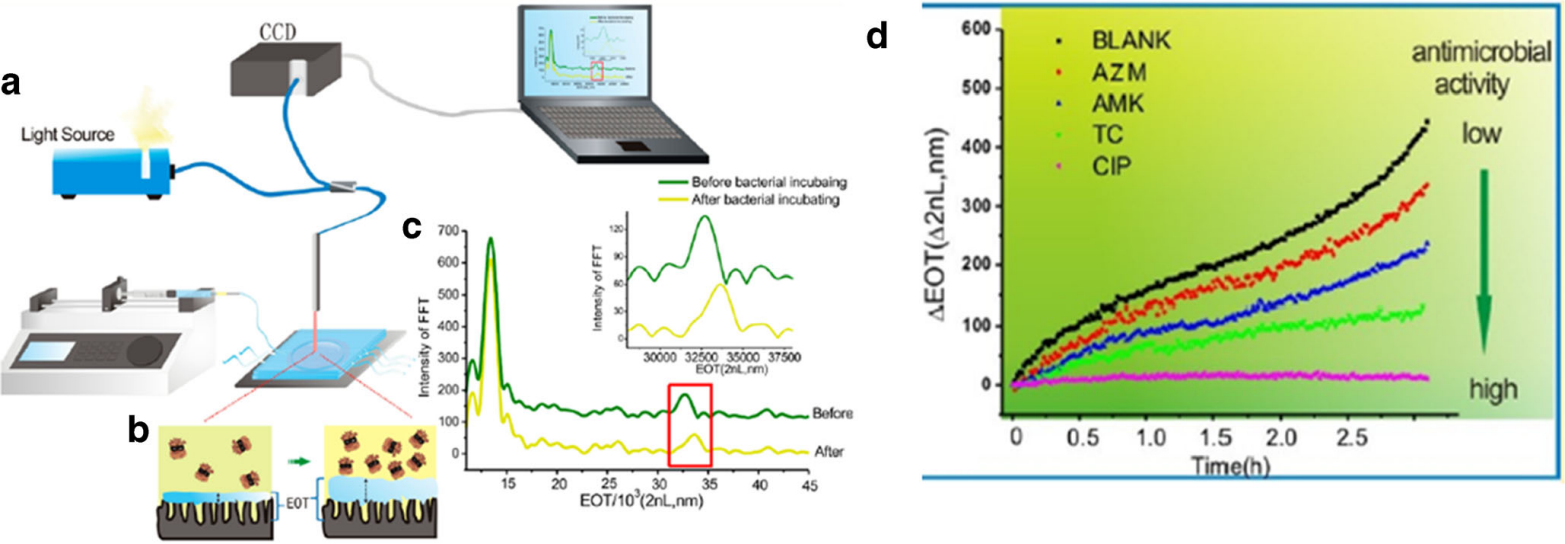
Microfluidic pH sensor. Schematic showing the experimental setup **a** and how the effective optical thickness (EOT) of pH-sensitive chitosan hydrogel changes as a consequence of bacterial growth **b**. **c** Fourier transform reflective interferometric spectroscopy (FT-RIFS) spectrum. The red box and inset highlight an EOT shift before and after incubation of the bacteria [78]. **d** EOT changes over time in *E. coli* cultures in the presence of different antibiotics (AZM, AMK, TC, CIP). Adapted with permission from Tang et al. [78]. Copyright (2017) American Chemical Society

Tang et al. [78] developed a microfluidic pH sensor in which a pH-responsive chitosan hydrogel is integrated in a microfluidic channel (Fig. 8b). Small amounts (10^8^ CFU/mL) of *E. coli* bacteria are constrained in these small channels of 0.8 μL volume [78]. As glucose-containing growth medium becomes increasingly acidic with an increasing amount of bacteria, since they produce organic acids while metabolising glucose, the pH of the medium can be directly related to the bacterial viability and growth. The swelling and shrinking of the chitosan hydrogel in response to pH changes can be assessed in real time by Fourier transform reflective interferometric spectroscopy (FT-RIFS). The effective optical thickness (EOT) is inversely related to the pH and can be quantified: higher EOT:pH ratios indicate greater bacterial activity and vice versa (Fig. 8d).

The microfluidic pH sensor has the major advantage in that a bacterial growth curve can be assessed within 2 h. As a major disadvantage, this method cannot be used with co-cultures, as eukaryotic cells would produce metabolites and, thereby, induce a pH change, independent of the bacterial growth. It remains to be determined whether this method can be used to assess antimicrobial resistance.

### Self-loading microfluidics device

**Fig. 9:**
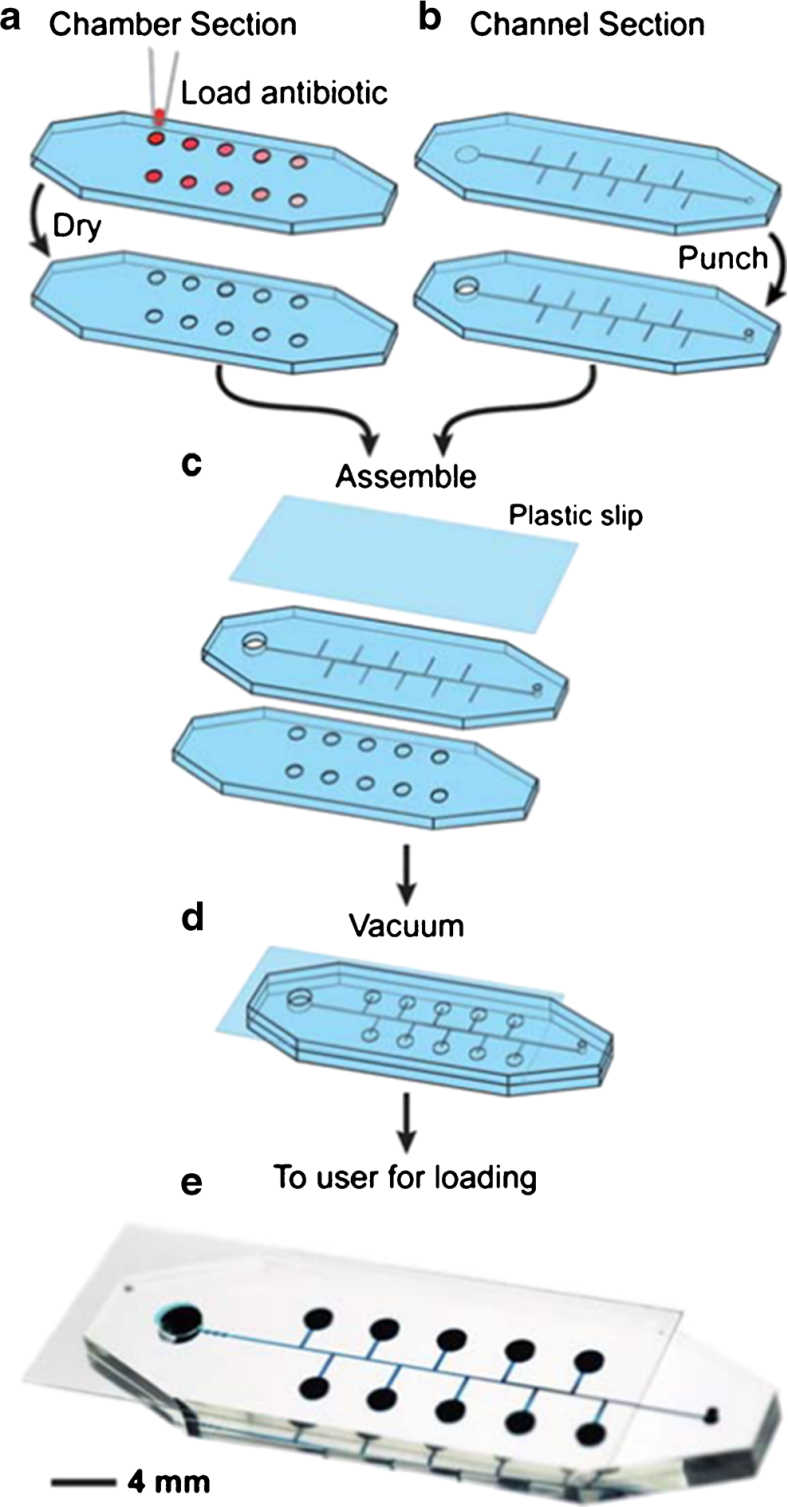
Fabrication and assembly of a self-loading microfluidics device. Chambers in the polydimethylsiloxane (PDMS) layer are filled with antibiotic solutions at different concentrations. Subsequently, the liquid is evaporated **a**. Holes are punched as inlet **b**. Assembly is done by aligning the chambers with the microfluidic channels and a plastic strip is applied to seal the construct **c**. Vacuum is applied **d** to finish it for the end user **e**. Reprinted with permission from Cira et al. [116]. Copyright (2017) Royal Society of Chemistry

Cira et al. [116] developed a portable microfluidics point-of-care device that determines the MIC of antibiotics. In this device, individual moulded polydimethylsiloxane (PDMS) dead-end chambers are covered with antibiotics and dried (Fig. 9). Prior to the bacterial injection, the degassed PDMS takes up gas from the environment and, thereby, creates a vacuum inside the channels connecting the chambers, creating a force that pulls the injected bacterial suspension into the chambers. This process was shown to result in comparable amounts of bacterial cells in each chamber. Upon entering the chambers, the bacterial suspension dissolves the dried antibiotics. As backflow is inhibited, no cross-contamination among the separate chambers can take place. In order to determine the MIC and distinguish between antibiotic-susceptible and -resistant bacteria, phenol red as well as glucose is added as a pH indicator to the cell suspension. The device is then incubated according to the CLSI standard for 18 h. As bacteria grow, they degrade glucose, which produces organic acids that decrease the pH of the solution, a reaction that can be easily observed by a calorimetric change. The MIC value is then defined as the lowest antibiotic concentration that does not change the colour from red to yellow. Resistant strains were identified by MIC values greater than 16-fold compared to the susceptible wild-type strain.

This self-loading device requires only a single pipetting step for testing several antibiotic concentrations simultaneously, which minimises the workload, the amount of reagents and equipment for readout. Furthermore, the chip enables testing of a variety of bacteria ranging from Gram-positive to Gram-negative ones. Its working is confirmed for *E. faecalis*, *Proteus mirabilis*, *Klebsiella pneumoniae* and *E. coli*. However, a major drawback is that the commercial test will offer only a standard number of antibiotics at certain concentrations, thereby leaving no experimental flexibility. Moreover, the determination of resistance is only done by measuring extreme concentrations compared to wild-type susceptible strains and does not take into account a method that could detect the mechanism of resistance. Despite the downsides, this and similar tools provide a new direction and opportunities in AST.

### Microdroplets

**Fig. 10:**
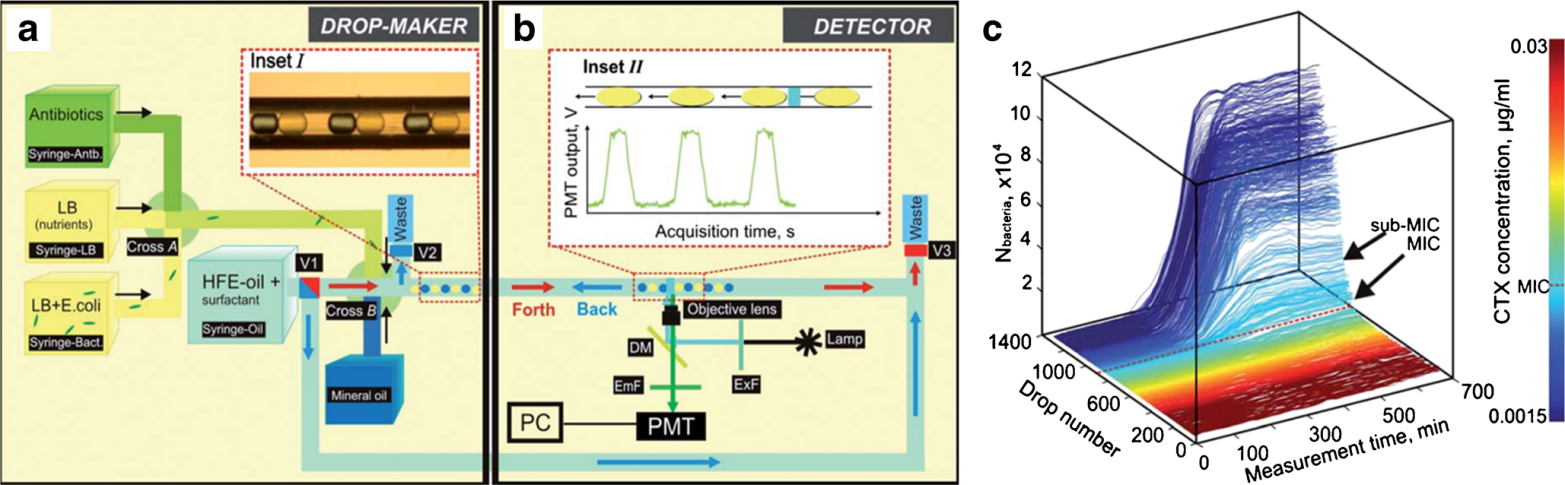
Schematic representation of the microfluidics droplet analyser, showing the drop maker **a** and detector compartment **b**, which analyses bacterial growth over time. **c** The bacterial growth is plotted for each antibiotic concentration (colour range) to determine the MIC. HFE = hydrofluoroether (HFE) oil, LB = syringe containing nutrients or bacteria, PMT = photomultiplier tube, DM = dichroic mirror, EmF = emission filter, ExF = excitation filter. Adapted with permission from Baraban et al. [117]. Copyright (2017) Royal Society of Chemistry

In the microdroplets method, microorganisms are embedded in inverse emulsion droplets, which are carried in a so-called carrier liquid (Fig. 10a) and possess a favourable cellular environment. Microdroplets are formed in tubes to avoid evaporation and contamination. Other tubes allow the addition of bacteria and antibiotics into the droplets in the process of droplet formation. As cellular changes alter the composition of the droplet, which can be an in- or outflux of water, the droplet can change shape. This can be detected and directly related to bioactivity of the microorganism. The technique can be used for many distinct bacterial types and strains, as bacteria-specific environments can be constructed within the droplets. It has been proven so far for several *E. coli*, *Saccharomyces cerevisiae* and *S. aureus* strains [111, 117–119]. With the artificial environment created in microdroplets, various experimental conditions can be easily manipulated [118]. Detection is fully automated and rapid. The MIC can be determined in less than 4 h [119]. Additionally, microdroplets can be used to assess susceptibility and resistance, as well as for single-cell analysis, by which the rate of nutrient consumption or the rate of metabolite production is determined. Baraban et al. [117] developed a millifluidic droplet analyser (MDA) for bacterial growth analysis (Fig. 10), which makes it possible to determine the growth rate, perform pharmacodynamics assays and quantify the MIC of antibiotics in bacterial cultures. Up to ≥ 10^3^ aqueous emulsion droplets with a volume of 100 nL each, containing approximately 10^6^ cells, can be formed and automatically detected by epifluorescent reporters that are expressed by the bacterial strains during droplet formation [118].

The major advantages of microdroplets are the possibility to fine-tune the antibiotic concentrations [117], the outstanding reproducibility of conditions within the droplets and the opportunity for high-throughput studies [111, 118, 119]. Furthermore, the single-cell method was found to be highly specific, as the metabolism of single cells can be observed. Long-term bacterial growth can be determined by scanning the same droplet array repeatedly. This technique may be limited when applied to bacterial species or strains that do not produce detectable osmotic changes or are unable to express a fluorescent reporter gene. The MDA disadvantageously depends on fluorescence, which requires special handling, always entails the risk of bleaching and can be affected by cellular lysis by antibiotics, as previously described [86]. While promising, this method requires further development and refinement to be reliably applied for AST.

## Osteoblast infection models

Osteoblast infection models are in vivo-like models which make use of co-cultures of bacteria and eukaryotic cells and have been developed in the past decade. Currently, such models are predominantly applied in research and no accepted clinical applications yet exist. By ensuring a certain degree of eukaryotic cell infection, the models can test the extra- and intracellular antimicrobial activity of compounds and assess the long-term effect of bacteria and antimicrobial compounds on human cells. This is necessary as various bacterial strains are likely to invade human cells. Readout generally requires fluorescence and microscopic tools, by which fluorescent intensity is related to cellular viability. Although these models are slow, they provide qualitative results on bacterial growth, offer insight into antimicrobial resistance mechanisms and provide the opportunity to test novel materials, such as bone graft substitutes.

### Human osteoblast infection model

Kreis et al. [120] set up a human osteoblast infection model in order to determine the activity of tigecycline against intracellular *S. aureus*, which is capable of invading human cells. Overnight bacterial cultures were added to a washed osteoblast culture and incubated for 30 min to ensure a multiplicity of infection (MOI) of 100, with an optical density of 1. Different antibiotic agents were then separately added to the cultures at a dosage that mimics the high concentration of intravenous administration. After 20 h and 40 h, the cells were washed, which destroys the osteoblasts and leaves behind only the intracellular bacteria. The bacteria were subsequently diluted and seeded on agar plates for CFU counting [120]. One major advantage of this model is that it resembles more closely an in vivo situation compared to other AST methods, as it includes the effect of antibiotics on internalised bacteria. However, it is difficult to assess whether this model can be extended to in vivo scenarios, as it has only been evaluated for *S. aureus*, which easily develops resistances to antibiotics [121]. Additionally, this model is time-consuming and requires sufficient cell and bacterial culture knowledge to properly interpret the results.

### Microfluidic titanium (Ti) alloy model

**Fig. 11:**
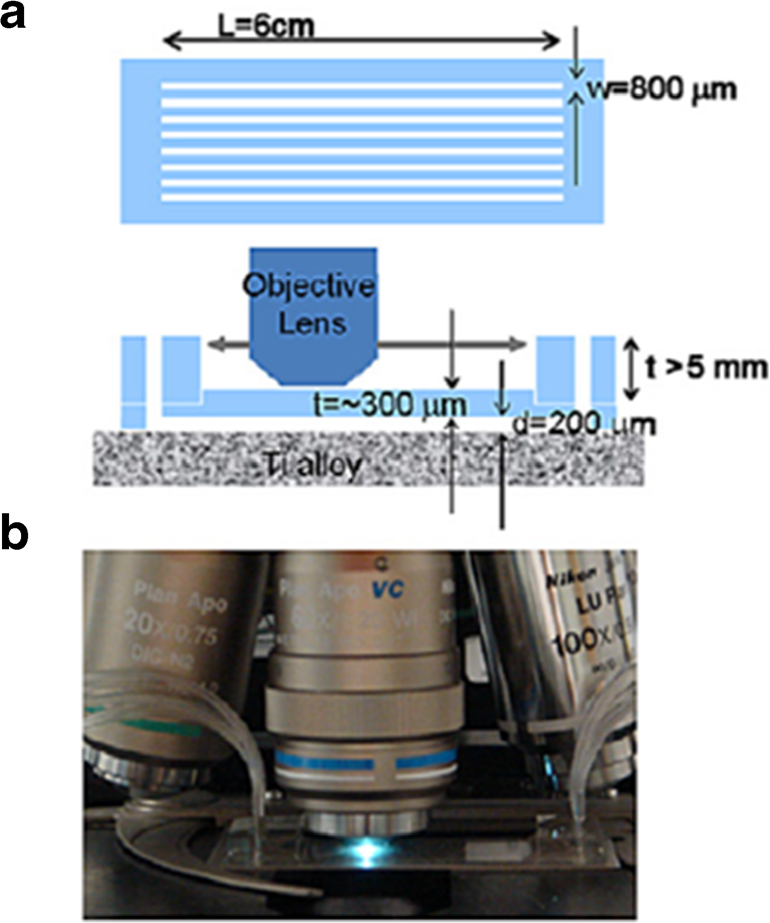
Design of the microfluidic titanium (Ti) alloy model to mimic osteoblast infection by *Staphylococcus epidermis*. **a** Schematic top and side representation of the model. **b** Image of the running model. Adapted from Lee et al. [122] with permission from Elsevier

In 2010, Lee et al. [122] developed a microfluidics-based model to mimic the in vivo infection of a titanium (Ti) alloy implant in a bony environment. To this end, they conducted a study on the effects of *S. epidermidis* on osteoblast cell adhesion and viability using a Ti alloy substrate inside a high-throughput poly(dimethylsiloxane) (PDMS) microfluidic network containing eight 10-μL channels (Fig. 11). The eight channels were simultaneously used for an osteoblasts mono-culture and co-culture of osteoblasts with small numbers (10^2^ or 10^5^ CFU/mL) of *S. epidermidis*. The latter included inoculation of Ti alloy surfaces with *S. epidermis* prior to the osteoblast addition. M-α-MEM (50% mixture of αMEM and Leibovitz’s L-15 medium) supplemented with 10 mM NaHCO_3_, 10 mM 4-(2-hydroxyethyl)-1-piperazineethanesulfonic acid (HEPES) and 10 vol% FBS was applied at a continuous flow rate. After a 25-h culture period, with effluent collection every 2 h, the channels were washed with PBS and stained with 30 μM propidium iodide. While the initial bacterial inoculation did not affect osteoblast viability, after 25 h, it was observed that osteoblasts were damaged by the fast bacterial proliferation. As *S. epidermis* is not considered pathogenic, a change in micro-environment, such as nutrient depletion and pH change, is assumed to have led to damaged osteoblasts [122].

The experimental setup of this microfluidics-based infection model—featuring osteoblasts, serum-based medium and a constant perfusion flow rate—mimics the in vivo situation representing infections related to bone implants. It has not yet been used for testing the effect of antibiotics, so its AST capabilities remain to be determined. This might be a challenge as with only the co-culture, osteoblasts were difficult to be kept alive after 25 h. Furthermore, the model does not support the analysis of intracellular bacteria, taken up by osteoblasts, which are usually responsible for recurrent infections. Nevertheless, its separated channels offer the possibility to test several antimicrobial agents or concentrations simultaneously, provided the inlets are connected to different sources. This interesting model will need improvement to give a more realistic and in vivo-like competition between osteoblasts and bacteria in “race for the surface”.

### Microfluidic 3D bone tissue model

**Fig. 12:**
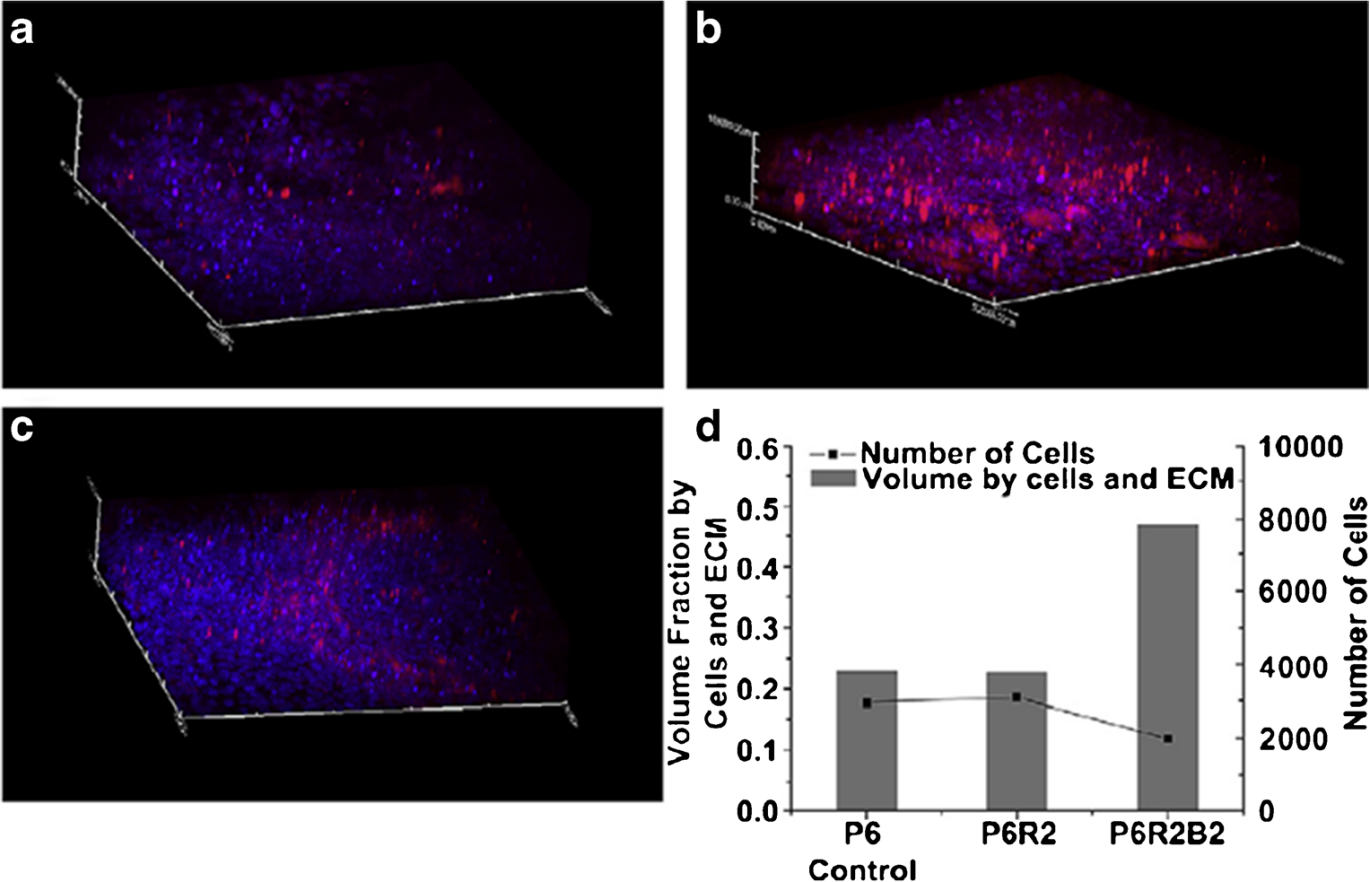
3D bone tissue model. **a**–**c** Two-photon microscopic images of the 3D bone tissue structure under the influence of the antibiotic- and calcium-eluting micropattern: **a** P6 control [pure poly(D,L-lactic-co-glycolic) acid (PLGA)]; **b** PGR2 (6% PLGA, 2% rifampicin); **c** PGR2B2 (6% PLGA, 2% rifampicin, 2% biphasic calcium phosphate (BCP)). **d** Quantification of the volume fraction of tissue (*left axis*) and number of cells (*right axis*) of three conditions. The presence of BCP in the PGR2B2 condition substantially increased the production of mineralised ECM. The antibiotic did not adversely affect osteoblast viability and tissue formation. Adapted from Lee et al. [123] with permission from Elsevier

Lee et al. [123] developed a 3D bone tissue model in order to evaluate wound healing and infection-preventing biomaterials. Osteoblasts, pre-cultured for 5 weeks, were added to a high-throughput model containing eight chambers made of bioresorbable poly(D,L-lactic-co-glycolic) acid (PLGA). 3D extracellular matrix formation by osteoblasts was observed in the chambers as early as after 5 days of culture, eventually leading to the formation of randomly oriented collagen fibres and calcium-rich mineral, after 5 weeks. Subsequently, the influence of inkjet-printed antibiotic- and calcium-eluting micropatterns (~100 nm rifampicin and ~100 nm biphasic calcium phosphate nanoparticles dispersed in the PLGA) within the eight chambers and the infection with a single *S. epidermis* strain was tested on the 3D tissue culture. The results were consistent with those obtained in their previously conducted 2D model [124]. The micropatterns were capable of killing the bacteria immediately and prevented biofilm formation. Neither 3D tissue formation nor osteoblast viability was adversely affected, as visualised by two-photon fluorescence and bright-field optical microscopy (Fig. 12).

This 3D bone tissue model has the major advantage in that it creates a more in vivo-like environment for bacterial susceptibility testing. The infection of osteoblasts and subsequent antibiotic treatment were shown to be successful in this model, as determined by osteoblast proliferation and survival. However, this model utilised osteoblasts and a single *S. epidermis* strain, and investigations with primary human cells as well as multiple bacterial species and strains are required to evaluate the model’s capabilities. Another disadvantage is that alkaline phosphatase and osteocalcin markers were difficult to measure due to the small amount of cells and effluents. Real-time monitoring of biomineralisation and bone tissue density could be a further improvement, as they would give much more reliable data on the efficacy and quality of 3D tissue culture [123]. While 3D models are considered more representative of in vivo systems, much more development is needed in order for such methods to be accepted as suitable replacements to traditional AST techniques.

## Discussion

Bacterial infections accompanying trauma surgery and total joint replacements have been an unresolved problem for decades. In order to determine optimal treatment and detect possible resistance of bacteria against antibiotics, AST methods have been developed and refined over the last century.

The first antimicrobial susceptibility methods on the market were various agar or broth dilution and disc diffusion techniques, which have become the gold-standard tests to which all other AST methods are currently compared during development, identification, validation and clinical trials [125]. Despite their simplicity, gold-standard techniques are still clinically applied with almost no changes since the first development, as they possess the major benefits of being cheap and not requiring any expensive equipment. Furthermore, as these techniques have been applied extensively, they are found to be very reliable in determining the antimicrobial effectivity and have been validated for a variety of bacterial strains. However, such techniques fail to provide insight into the mechanism of antimicrobial resistance, are relatively time-consuming and require knowledge of working with the specific bacteria.

Newer techniques, including mechanical, optical and microfluidics methods, were found to be suitable for single-cell analysis, subsequent quantification and for determining underlying resistance mechanisms. In addition, they provide much faster MIC values and depiction of antimicrobial resistance, even at low bacterial concentrations. Another major benefit is that these systems are isolated, which creates a much safer work environment. Microfluidics and microdroplets methods qualify as high-throughput systems, as they enable the fine-tuning of antibiotic concentrations and other complex conditions in a reproducible manner. However, most of these methods have only been proven to function for one type of bacteria and, therefore, need validation for other strains, as well as the ability to analyse bacterial mixtures. Furthermore, all systems require preparation of clinical samples or depend on fluorescence, which augments the workload due to special handling.

Compared to microfluidics methods, the osteoblast infection models extend the possibility to create a more in vivo-like infective situation. This allows for a more in-depth research of the bacterial–human cell interaction, internalisation of bacteria into host cells and, furthermore, the mechanism of resistance for an antimicrobial compound, as actual tissue is grown. Unfortunately, these models are currently only suitable for research rather than clinical application due to their intensive mode of use. Precise control of the bacteria–cell–antibiotic ratio and further culture conditions are required in order to mimic a naturally occurring infection. Furthermore, little to no validation is performed of these models for AST and such tissue models require weeks of culture. However, they show great promise in becoming of important diagnostic value in implant-related infections.

Although not discussed within this review, biofilm models feature prominently in AST. Planktonic and biofilm bacteria were found to be affected differently by antibiotics and, therefore, need different models as well [126]. Actual biofilm models provide better information on resistance mechanism in bacterial cell aggregations. One step further in designing realistic infection models would then be the implementation of biomaterials such as bone graft materials into biofilm models to imitate host tissue infection. These models would be extremely useful as, first, more realistic infection models would be created and, second, novel, smart bone graft substitutes [127] and implant coatings could be tested for their antimicrobial characteristics.

## Conclusion

In conclusion, this review of the existing antimicrobial susceptibility testing (AST) methods has shown that it is difficult to develop a system that combines the advantages of being precise, versatile, inexpensive and easy to perform. The selection of one AST method over another depends on the application: clinical needs for analysing patient material are different from what is required to answer important fundamental research questions. A method applicable to both environments is not very likely to be developed. Even if state-of-the-art models such as osteoblast infection or 3D tissue models can mimic in vivo-like situations to a certain extent, their current clinical value is low. This is due to their time-consuming process, requirement of sufficient cell and bacterial knowledge, and the fact that their application is limited to determining the microbial and antimicrobial coating and implant interaction. Osteoblast infection models have to be expanded towards multiple bacteria and strains, the introduction of biomaterials, as well as reduce their time consumption to be of sufficient clinical value. An exception are microfluidics methods, which show great potential for both research and clinical application. With easy readouts and high-throughput, they can be useful in the clinic, while offering the possibility to perform in-depth mechanistic study, relevant to fundamental research. Continued validation and optimisation of these new methods should offer robust AST options. In particular, with recent reports of increased antimicrobial resistance, there is an urgency to develop and implement methods that analyse recurrent infections induced by intracellular bacteria. These could be biofilm models including biomaterials in the future.

## Appendix

**Table 1:** Summary of current antimicrobial susceptibility testing (AST) methods and their features. Gold-standard methods are approved and mostly applied in clinics and as control/reference in research (X). Research AST models are partly commercially available and partly proof-of-concept studies. Note that this table is a qualitative assessment to summarise the findings of this review. YOP is defined as the year of publication or as the year of introduction_1_ of a gold-standard method. EOP is defined by the complexity of handling, the complexity of the setup and required knowledge. Methods can be either manual (M), automated (A) or both (M/A). UNK = unknown

Method		YOP	Mean time to results	Main application		EOP	M/A	Results		Determines resistance	Validated with...		
				Clinic	Research			Qualitative	Quantitative		Bacteria	Cells	Antibiotics
Gold-standard	Agar dilution	1929_1_	24 h	X	(X)	Easy	M		X	No	X		X
	Chromogenic agar	2009	18–24 h	X	(X)	Easy	M		X	Yes	X		X
	Broth macrodilution	1971_1_	24 h	X	(X)	Easy	M		X	No	X		X
	Disc diffusion	1959_1_	18–24 h	X	(X)	Easy	M	X		Yes	X		X
	Etest	2010	24 h	X	(X)	Easy	M		X	No	X		X
	Microdilution	1977_1_	24 h	X	(X)	Easy	M		X	No	X		X
	Microcalorimetry	1973_1_	4–5 h	X	(X)	Easy	M		X	Yes	X		X
	PCR-based methods	1992–2016	2 h	X	(X)	Moderate	M/A		X	Yes	X		X
Mechanical	Asynchronous magnetic bead rotation (AMBR) sensor	2014	6–24 h		X	Complex	M		X	UNK	X		X
	Single-cell AMBR sensor	2011	2 h		X	Complex	M	X		UNK	X		X
	Surface acoustic wave sensor	2007	7 h		X	Moderate	M		X	No	X		
	Microbial cell weighting with vibrating cantilevers	2010	20 min to 2 h		X	Complex	M		X	Yes	X		X
Optical	Optical density measurement	1975–2014	2–3 h		X	Easy	M/A		X	Yes	X		X
	Biomimetic polymer sensor	2006	6–18 h		X	Easy	M		X	Yes	X		X
	Flow cytometry	1997–2014	2 h		X	Moderate	M/A		X	Yes	X		X
	Image analysis software	2006–2015	UNK		X	Complex	M/A	X	X	UNK	X		X
	Optical tweezers	2015	100–200 s		X	Complex	M		X	No	X		
	Raman spectroscopy	2004–2010			X	Complex	M		X		X		X
	MALDI-TOF MS	2005–2013	12–24 h	X	X	Complex	M/A		X	Yes	X		X
	Automated systems	1975	UNK	X	X	Moderate	A		X	Yes	X		X
Microfluidics	Microfluidic agarose channels	2013	3–4 h		X	Moderate	M		X	Yes	X		X
	Microfluidic pH sensor	2013	2 h		X	Moderate	M/A		X	UNK	X		X
	Self-loading microfluidic device	2012	18		X	Easy	M	X		Yes	X		X
	Microdroplets	2011–2016	< 4 h		X	Complex	M/A		X	Yes	X		X
Osteoblast infection models	Human osteoblast infection	2013	> 20 h		X	Moderate	M		X	Yes	X	X	X
	Microfluidics Ti alloy model	2010	25 h		X	Complex	M	X	X	UNK	X	X	
	Microfluidics 3D bone tissue	2012	5 weeks		X	Complex	M	X		Yes	X	X	X
